# Plant growth promoting rhizobia: challenges and opportunities

**DOI:** 10.1007/s13205-014-0241-x

**Published:** 2014-08-03

**Authors:** Subramaniam Gopalakrishnan, Arumugam Sathya, Rajendran Vijayabharathi, Rajeev Kumar Varshney, C. L. Laxmipathi Gowda, Lakshmanan Krishnamurthy

**Affiliations:** International Crops Research Institute for the Semi-Arid Tropics (ICRISAT), Patancheru, 502 324 Andhra Pradesh India

**Keywords:** Rhizobium, PGPR, Biocontrol, Stress, Heavymetal, Co-inoculation

## Abstract

Modern agriculture faces challenges, such as loss of soil fertility, fluctuating climatic factors and increasing pathogen and pest attacks. Sustainability and environmental safety of agricultural production relies on eco-friendly approaches like biofertilizers, biopesticides and crop residue return. The multiplicity of beneficial effects of microbial inoculants, particularly plant growth promoters (PGP), emphasizes the need for further strengthening the research and their use in modern agriculture. PGP inhabit the rhizosphere for nutrients from plant root exudates. By reaction, they help in (1) increased plant growth through soil nutrient enrichment by nitrogen fixation, phosphate solubilization, siderophore production and phytohormones production (2) increased plant protection by influencing cellulase, protease, lipase and β-1,3 glucanase productions and enhance plant defense by triggering induced systemic resistance through lipopolysaccharides, flagella, homoserine lactones, acetoin and butanediol against pests and pathogens. In addition, the PGP microbes contain useful variation for tolerating abiotic stresses like extremes of temperature, pH, salinity and drought; heavy metal and pesticide pollution. Seeking such tolerant PGP microbes is expected to offer enhanced plant growth and yield even under a combination of stresses. This review summarizes the PGP related research and its benefits, and highlights the benefits of PGP rhizobia belonging to the family Rhizobiaceae, Phyllobacteriaceae and Bradyrhizobiaceae.

## Introduction

Imbalance in nitrogen (N) cycling, nutritional status, physical and biological properties of soil, incidence of pests and diseases, fluctuating climatic factors and abiotic stresses are the interlinked contributing factors for reduced agricultural productivity. Agricultural sustainability, food security and energy renewability depends on a healthy and fertile soil. However, rapid acceleration of desertification and land degradation by numerous anthropogenic activities leads to an estimated loss of 24 billion tons of fertile soil from the world’s crop lands (FAO 2011). The intensity of such degradation can be realized by the extent of highly degraded (25 %) and slightly/moderately degraded (36 %) lands, while only 10 % of land is listed to be improving all though high level use of agricultural chemicals have increased the productivity of available limited lands, high energy and environmental costs associated with their use necessitate the search for alternative methods of soil fertility and pest management. Recent estimations indicate that by 2030, the increasing population growth and changing consumption patterns would increase the demand for food by at least 50 %, energy by 45 % and water by 30 % (IFPRI 2012). These expectations cannot be met sustainably unless the soil fertility and productivity has been restored in the already degraded lands. A reversal of the decline in soil health is a possibility through the use of green and farm yard manures, composts and crop residues and by crop management options, such as natural fallow, intercropping, relay cropping, cover crops, crop rotations and dual purpose legumes. Among these practices, legumes are the well-acknowledged builders and restorers of soil fertility, primarily through their association with symbiotic nitrogen fixation.

Use of microbial agents for improving agricultural productions, soil and plant health had been practiced for centuries. By the end of the ninenteenth century, the practice of mixing natural soil with seeds became a recommended method of legume inoculation. Rhizospheric soil, inhabited and influenced by the plant roots, is usually rich in nutrients when compared to the bulk soil, due to the accumulation of numerous amino acids, fatty acids, nucleotides, organic acids, phenols, plant growth regulators/promoters, putrescine, sterols, sugars and vitamins released from the roots by exudation, secretion and deposition. This results in enrichment of microorganisms (10- to 100-folds than the bulk soil) such as bacteria, fungus, algae and protozoa, among which bacteria influence the plant growth in a most significant manner (Uren 2007). Such rhizobacteria were categorized depending on their proximity to the roots as (1) bacteria living in soil near the roots (rhizosphere) (2) bacteria colonizing the root surface (rhizoplane) (3) bacteria residing in root tissue (endophytes), inhabiting spaces between cortical cells and (4) bacteria living inside cells in specialized root structures, or nodules, which includes two groups—the legume associated rhizobia and the woody plant associated *Frankia* sp. (Glick 1995). Bacteria that belong to any of these categories and promote plant growth either directly (nitrogen fixation, phosphate solubilization, iron chelation and phytohormone production) or indirectly (suppression of plant pathogenic organisms, induction of resistance in host plants against plant pathogens and abiotic stresses), are referred as plant growth promoting rhizobacteria (PGPR). Vessey (2003) preferred to categorize the bacteria that belong to the above mentioned first three groups as extracellular PGPR (ePGPR) and the fourth group as intracellular PGPR (iPGPR). This ePGPR includes the genera *Bacillus, Pseudomonas, Erwinia, Caulobacter, Serratia, Arthrobacter, Micrococcus, Flavobacterium, Chromobacterium, Agrobacterium, Hyphomycrobium* and iPGPR includes the genera *Rhizobium, Bradyrhizobium, Sinorhizobium, Azorhizobium, Mesorhizobium* and *Allorhizobium.*

Research on exploring the potential of such PGPR has been reviewed periodically by many researchers (Bhattacharyya and Jha 2012; Gray and Smith 2005; Johri et al. 2003; Lugtenberg and Kamilova 2009). There are many reviews focusing on both ePGPR and iPGPR. However, we intend to provide a detailed review on iPGPR, the rhizobia that belong to the families Rhizobiaceae (excluding the *Frankia* sp.), Bradirhizobiaceae and Phyllobacteriaceae, having unique association with root nodules of legumes and induce plant growth in many ways and improving sustainability in agriculture. Similar review on the capacity of rhizobia in inducing the plant growth of nonleguminous plants has been published by Mehboob et al. (2012).

In Rhizobiaceae family, the constituents increased considerably from 8 in the year 1980 to 53 in 2006 (Willems 2006). Dispersion of host plants to new geographical locations might serve as a major source for these new rhizobia species. Still, increasing number of rhizobial species is expected because of following reasons. Only 57 % of 650 genera of leguminous plants have been studied for nodulation. Exploration of large number of legume species can potentially lead to the identification of many more rhizobial species. Recent advancements in the taxonomic research with the aid of specific molecular tools are another reason. So, identification and exploration of such potential rhizobia with plant growth promoting properties will be useful for sustainable agriculture.

## Plant growth promoting traits of rhizobia

Plant growth promotion by Rhizobia can be both direct as well as indirect. Both these types of promotions are discussed as follows:

### Direct promotions

#### Nitrogen fixation

Nitrogen (N) is required for synthesis of nucleic acids, enzymes, proteins and chlorophyll and hence it is a vital element for plant growth. Although 78 % of the atmospheric air is N, this gaseous form is unavailable for direct assimilation by plants. Currently a variety of industrial N fertilizers is used for enhancing agricultural productivity. However, economic, environmental and renewable energy concerns dictate the use of biological alternatives. Biological nitrogen fixation (BNF) is a process of converting atmospheric N into plant assimilable N such as ammonia through a cascade of reactions between prokaryotes and plants with the use of complex enzyme systems (Wilson and Burris 1947). BNF accounts for about 65 % of N currently used in agriculture. Legumes are BNF capable and meet their own N needs. Major part of N fixed by legumes is harvested as grains, while the soil and the succeeding crops also get benefitted by N in the form of root and shoot residues. Legume crops substantially reduce the N requirement from external sources (Bhattacharyya and Jha 2012). However, N fixation efficiency of legumes varies, and depends on the host genotype, rhizobial efficiency, soil conditions, and climatic factors. Reported quantum of nitrogen fixation ranged from 126 to 319 kg N ha^−1^ in groundnut, 33 to 643 kg N ha^−1^ in soybean, 77 to 92 kg N ha^−1^ in pigeonpea, 25 to 100 kg N ha^−1^ in cowpea, 71 to 74 kg N ha^−1^ in green gram and 125 to 143 kg N ha^−1^ in black gram (Peoples and Craswell 1992). Crops like wheat, rice, sugarcane and woody species have also the capacity to fix atmospheric N using free living or associative diazotrophs like *Cyanobacteria*, *Azospirillum*, *Azocarus* etc. However, the contribution of legume-rhizobia symbiosis (13–360 kg N ha^−1^) is far greater than the non-symbiotic systems (10–160 kg N ha^−1^) (Bohlool et al. 1992). The symbiotic N contribution is also reported to benefit the cereal crops, such as maize, rice, wheat and sorghum with a relative yield increase of 11–353 % (Peoples and Cranswell 1992).

Rhizobia can be used as inoculants for enhanced N fixation and studies demonstrated their predominance in nodules for 5–15 years after initial inoculation (Lindström et al. 1990), and confirming that they are effective colonizers persisting in soil for many years in the absence of their host (Sanginga et al. 1994). Still, BNF systems can be realized only through analysis and resolution of major constraints to their optimal performance in the field, their adoption and use by the farmers. The constraints include environmental, biological, methodological, production level and sociocultural aspects (Bohlool et al. 1992).

BNF ability, N self sustainability and protein-rich grains of legumes require high energy and productivity tradeoffs (Hall 2004). Hence, improving yield potential of BNF capable legumes, to a level of cereals, is considered difficult. Legumes do not establish a rapid crop ground coverage leading to low intercepted photon (radiation) use efficiency and a low proportion of carbohydrate that is partitioned to the grain. In addition, this area of research had attracted less plant breeding attention till now. The energy costs of biochemical pathways for the production of proteins and lipids are far greater than that of carbohydrates. This explains why the protein-rich legumes lack behind in yield potential compared to cereals. Production of proteins require 2.5 and lipids 3.0 units of photosynthates (glucose), while a mere 1.2 units are required for the carbohydrates (Penning de Vries et al. 1983) and such a high energy requirement for protein synthesis and accumulation in seeds increases the amount of photosynthates requirement thereby reducing the productivity potential of legumes and oil seeds. The seed biomass production efficiency of legumes is shown to be lower (0.66) per unit of photosythates required as compared to 0.72 of cereals (Sinclair and de Wit 1975). Also, legume seeds (26 mg g^−1^ seed) need double the requirement of nitrogen compared to cereals (13 mg g^−1^ seed) which is one more limiting factor for grain yield productivity. In addition, legumes need to be BNF-capable spending large amount of energy in this symbiotic relationship contributing both for the current yield and for enriching the soil by 30–40 kg N for every ton of plant biomass productivity (Peoples et al. 2009). Thus, not only the BNF requires energy, but also it acts as a limiting factor to yields when conditions are uncongenial.

Nitrogenase, a major enzyme involved in the nitrogen fixation has 2 components: (1) dinitrogenase reductase, the iron protein and (2) dinitrogenase (metal cofactor). The iron protein provides the electrons with a high reducing power to dinitrogenase which in turn reduces N_2_ to NH_3_. Depending on the availability of metal cofactor, three types of N fixing systems have been identified (1) Mo-nitrogenase (2) V-nitrogenase and (3) Fe-nitrogenase. Complexity of nitrogen fixation can be clearly understood by the contribution of several gene clusters for (1) nitrogen fixation (*nifHDK*—nitrogenase, *nifA*, *fixLJ*, *fixK*—transcriptional regulator, *nifBEN*—biosynthesis of the Fe-Mo cofactor, *fixABCX*—electron transport chain to nitrogenase, *fixNOPQ*—cytochrome oxidase, *fixGHIS*—copper uptake and metabolism, *fdxN*—ferredoxin) (2) nodulation (*nodA*—acyltransferase, *nodB*—chitooligosaccharide deacetylase, *nodC*—*N*-acetylglucosaminyltransferase, *nodD*—transcriptional regulator of common nod genes, *nodIJ*—Nod factors transport, *nodPQ*, *nodX*, *nofEF*, NOE—synthesis of Nod factors substituents, *nol* genes—several functions in synthesis of Nod factors substituents and secretion); and (3) other essential elements (*exo*—exopolyssacharide production*, hup*—hydrogen uptake*, gln*—glutamine synthase*, dct*—dicarboxylate transport*, nfe*—nodulation efficiency and competitiveness*, ndv*—β-1,2 glucan synthesis*, pls*—lipopolysaccharide production) (Laranjo et al. 2014). Another study reported the coexistence of symbiosis and pathogenicity determining genes in *Rhizobium* (Velázquez et al. 2005). This coexistence enables the induction of nodules depending on plant species. Although BNF is an energy expensive process, it is the only process through which the atmospheric N is converted to plant usable organic N making the greatest quantitative impact on N cycle. Legume–rhizobia (*Rhizobium*/*Bradyrhizobium*/*Mesorhizobium*) symbiosis is a cheaper source of N and an effective agronomic practice ensuring adequate supply of N than the application of fertilizer-N. However, various environmental factors limit nitrogen fixation, such as soil moisture deficiency, osmotic stress, extremes of temperature, soil salinity, soil acidity, alkalinity, nutrient deficiency, overdoses of fertilizers and pesticides; since all these soil and environmental factors affect the survival and infectivity rate of rhizobia—an important driver for BNF (Zahran 1999). Recent research is focused to identify rhizobial strains with resistance to these environmental stresses and explore their potentiality under field conditions. Details of such rhizobia have been discussed in later parts of this review.

Nitrification is an important process in nitrogen cycle in which ammonia is converted to nitrite and nitrate by nitrifying bacteria such as *Nitrosomonas* and *Nitrobacter*. The nitrification products are vulnerable to leaching and denitrification and an estimated 45 % of applied fertilizer is lost by leaching (Jarvis 1996) and 10–30 % by denitrification (Parker 1972). Therefore, a reduced rate or inhibition of nitrification provides enough time to plant for assimilation of fixed N. Plants also produce secondary metabolites such as phenolic acids and flavonoids for inhibiting nitrification. The natural ability of plants to suppress nitrification is not currently recognized or utilized in agricultural production (Subbarao et al. 2006). However, they have no effects on other soil microbial community. For example, it had been demonstrated that nitrification inhibitor produced by *B. humidicola* as root extracts were seen to inhibit nitrifying bacteria, with no adverse effects on other soil microorganisms such as *Azospirillum lipoferum*, *R. leguminosarum* and *Azotobacter chroococcum* (Gopalakrishnan et al. 2009). This work also demonstrated that, this inhibitory effect vary with the soil type. Nitrification and denitrification remain to be the only known biological processes that generate nitrous oxide (N_2_O), a powerful greenhouse gas contribute to global warming. Biological nitrification inhibition is seen as a major mitigation process towards global warming besides improving N recovery and N use efficiency of agricultural systems (Subbarao et al. 2012).

#### Phosphate solubilizers

After nitrogen, phosphorus (P) is the most limiting nutrient for plant growth. It exists in both inorganic (bound, fixed, or labile) and organic (bound) forms and the concentration depends on the parental material. The concentration had been shown to range from 140 ppm in carbonate rocks to more than 1,000 ppm in volcanic materials (Gray and Murphy 2002). Although the parent material has a strong control over the soil P status of terrestrial ecosystems (Buol and Eswaran 2000), the availability of P to plants is influenced by pH, compaction, aeration, moisture, temperature, texture and organic matter of soils, crop residues, extent of plant root systems and root exudate secretions and available soil microbes. Soil microbes help in P release to the plants that absorb only the soluble P like monobasic (H_2_PO_4_^−^) and dibasic (H_2_PO_4_^2−^) forms (Bhattacharyya and Jha 2012). Although the P fertilizer provides the plants with available form of P, excessive application of them is not only expensive, but also damaging to environment.

Phosphorus accounts for about 0.2–0.8 % of the plant dry weight, but only 0.1 % of this P is available for plants from soil (Zhou et al. 1992). The soil solution remains to be the main source of P supply to plants. The P content of agricultural soil solutions are typically in the range of 0.01–3.0 mg P L^−1^ representing a small portion of plant needs. The rest must be obtained from the solid phase through intervention of biotic and abiotic processes where the phosphate solubilizing activity of the microbes has a role to play (Sharma et al. 2013). Rhizobia, including *R. leguminosarum, R. meliloti, M. mediterraneum, Bradyrhizobium* sp. and *B. japonicum* (Afzal and Bano 2008; Egamberdiyeva et al. 2004; Rodrigues et al. 2006; Vessey 2003) are the potential P solubilizers. These bacteria synthesize low molecular organic acids which acts on inorganic phosphorous. For instance, 2-ketogluconic acid with a phosphate-solubilizing ability has been identified in *R. leguminosarum* (Halder et al. 1990) and *R. meliloti* (Halder and Chakrabarty 1993). Sometimes mineralization of organic P takes place by several enzymes of microbial origin, such as acid phosphatases (Abd-Alla 1994a, b), phosphohydrolases (Gügi et al. 1991), phytase (Glick 2012; Richardson and Hadobas 1997), phosphonoacetate hydrolase (McGrath et al. 1998), D-α-glycerophosphatase (Skrary and Cameron 1998) and C–P lyase (Ohtake et al. 1996). Some bacterial strains are found to possess both solubilization and mineralization capacity (Tao et al. 2008). Importance of this P solubilizing capacity in enhancing plant growth by *M. mediterraneum* has been demonstrated in chickpea and barley plants (Peix et al. 2001).

#### Siderophore formation

Iron, a typical essential plant micronutrient, is present in soils ranging from 0.2 to 55 % (20,000–550,000 mg/kg) with a significantly different spatial distribution. Iron can occur in either the divalent (ferrous or Fe^2+^) or trivalent (ferric or Fe^3+^) states which is determined by the pH and Eh (redox potential) of the soil and the availability of other minerals (e.g., sulphur is required to produce FeS_2_ or pyrite) (Bodek et al. 1988). Under aerobic environments, iron exists as insoluble hydroxides and oxyhydroxides, which are not accessible to both plants and microbes (Rajkumar et al. 2010). Generally bacteria have the ability to synthesis low molecular weight compounds termed as siderophores capable of sequestering Fe^3+^. These siderophores are known to have high affinity for Fe^3+^, and thus makes the iron available for plants. The siderophores are water soluble and are of two types viz. extracellular and intracellular. Fe^3+^ ions are reduced to Fe^2+^ and released into the cells by gram positive and negative rhizobacteria. This reduction results in destruction/recycling of siderophores (Rajkumar et al. 2010). Siderophores can also form stable complex with heavy metals such as Al, Cd, Cu etc. and with radionucleides including and U and NP (Neubauer et al. 2000). Thus, the siderophore producing bacteria can relieve plants from heavy metal stress and assist in iron uptake. Rhizobial species, such as *R. meliloti, R. tropici, R. leguminosarum* bv. *viciae, R. leguminosarum* bv. *trifolii, R. leguminosarum* bv. *phaseoli, S. meliloti* and *Bradyrhizobium sp.* are known to produce siderophores (Antoun et al. 1998; Arora et al. 2001; Carson et al. 2000; Chabot et al. 1996).

#### Phytohormone production

Phytohormones are the substances that stimulate plant growth at lower/equal to micromolar concentrations. These include indole-3-acetic (IAA) acid (auxin), cytokinins, gibberellins and abscisic acid.

*Indole-3-acetic acid (IAA)—*IAA is the foremost phytohormone that accelerates plant growth and development by improving root/shoot growth and seedling vigor. IAA is involved in cell division, differentiation and vascular bundle formation and an essential hormone for nodule formation. It has been estimated that 80 % of bacteria isolated from the rhizosphere can produce IAA (Patten and Glick 1996). The salient ones are *A. caulinodans, B.japonicum, B. elkanii, M. loti, R. japonicum, R. leguminosarum, R. lupine, R. meliloti, R. phaseoli, R. trifolii* and *Sinorhizobium spp*. (Afzal and Bano 2008; Antoun et al. 1998; Biswas et al. 2000; Boiero et al. 2007; Chi et al. 2010; Chandra et al. 2007; Dazzo et al. 2005; Naidu et al. 2004; Senthilkumar et al. 2009; Yanni et al. 2001; Weyens et al. 2009). IAA production in rhizobium takes place via indole-3-pyruvic acid and indole-3-acetic aldehyde pathway. On inoculation of *R. leguminosarum* bv*. viciae,* 60-fold increase in IAA was observed in the nodules of vetch roots (Camerini et al. 2008). One of the highest productions of IAA had been reported with the inoculation with *B. japonicum*-SB1 with *B. thuringiensis*—KR1 (Mishra et al. 2009). Co-inoculating *Pseudomonas* with *R. galegae* bv. *orientalis* had shown to produce IAA that had contributed to increases in nodule number, shoot and root growth and nitrogen content. Both environmental stress factors (acidic pH, osmatic and matrix stress and carbon limitation) and genetic factors (auxin biosynthesis genes and the mode of expression) were shown to influence the biosynthesis of IAA (Spaepen et al. 2007; Spaepen and Vanderleyden 2011).

*Cytokinins*—Cytokinin stimulates plant cell division and in some instances root development and root hair formation (Frankenberger and Arshad 1995). It is documented that 90 % of rhizospheric microorganisms are capable of releasing cytokinins and about 30 growth-promoting compounds of the cytokinin group has been identified from microbial origin (Nieto and Frankenberger 1990, 1991). *Rhizobium* strains are also reported as the potent producers of cytokinins (Caba et al. 2000; Senthilkumar et al. 2009).

*Gibberellins*—Gibberellins, the plant hormones responsible for stem elongation and leaf expansion has been denoted as GA1 to GA89 depending on the approximate order of their discovery. It is also believed that certain types of dwarfness are due to gibberellin deficiency, but it has no effect on roots. Application of gibberellins is known to promote bolting of the plants, parthenocarpy in fruits, increase fruit size and number of buds and break down the tuber dormancy. Gibberellins also help in seed germination as in the case of lettuce and cereals and control flowering and sex expression of flowers. Many PGP microbes are reported to produce gibberellins (Dobbelaere et al. 2003; Frankenberger and Arshad 1995) including *Rhizobium*, *S. meliloti* (Boiero et al. 2007).

*Abscisic acid*—Abscisic acid in plants is synthesized partially in the chloroplasts and the whole biosynthesis primarily occurs in the leaves. The production of abscisic acid is accentuated by stresses such as water deficit and freezing temperatures. It is believed that biosynthesis occurs indirectly through the production of carotenoids. The transport of abscisic acid can occur in both xylem and phloem tissues and can also be translocated through paranchyma cells. The movement of abscisic acid in plants does not exhibit polarity like auxins (Walton and Li 1995). Abscisic acid was reported to stimulate the stomatal closure, inhibit shoot growth while not affecting or even promoting root growth, induce seeds to store proteins and in dormancy, induce gene transcription for proteinase inhibitors and thereby provide pathogen defense and counteract with gibberellins (Davies 1995; Mauseth 1991). *Rhizobium* sp. and *B. japonicum* had been reported to produce abscisic acid (Boiero et al. 2007; Dobbelaere et al. 2003).

##### 1-aminocyclopropane-1-carboxylic acid (ACC) deaminase

ACC deaminase is a member of a large group of enzyme that utilizes vitamin B6 and considered to be under tryptophan synthase family. Rhizobia has the ability to uptake ACC and convert it into α-ketobutyrate and NH_3_. This is used as a source of carbon and nitrogen. Hence, on inoculation of rhizobia producing ACC deaminase, the plant ethylene levels are lowered and result in longer roots providing relief from stresses, such as heavy metals, pathogens, drought, radiation, salinity, etc. Strains, such as *R. leguminosarum* bv. *viciae*, *R. hedysari, R. japonicum, R. gallicum, B. japonicum, B. elkani, M. loti* and *S. meliloti* had been known to produce ACC deaminase (Duan et al. 2009; Hafeez et al. 2008; Kaneko et al. 2000; Ma et al. 2003a, b, 2004; Madhaiyan et al. 2006; Okazaki et al. 2004; Sullivan et al. 2002; Uchiumi et al. 2004). IAA producing bacteria are reported to produce high levels of ACC and known to inhibit ethylene levels (Glick 2014). Inoculation with these bacteria had shown to promote root elongation, shoot growth, enhanced rhizobial nodulation and minerals uptake (Glick 2012). It had also been shown that the rhizobia producing ACC deaminase are also efficient nitrogen fixers. The structural gene of ACC deaminase (*acds*) in *Mesorhizobium* sp. is under the control of *nif* promoter (Nascimento et al. 2012) which generally controls the *nif* gene responsible for nitrogen fixation.

### Indirect growth promotions

There are many indirect ways through which rhizobia act as plant growth promoters with their biocontrol properties and induction of systemic resistance against phytopathogens and insect pests. PGP organisms have the ability to produce many active principles for biocontrol of various phytopathogens with antibiosis production. This includes (1) production of antibiotics such as 2,4-diacyetyl phloroglucinol (DAPG), kanosamine, phenazine-1-carboxylic acid, pyoluteorin, neomcycin A, pyrrolnitrin, pyocyanin and viscosinamide. Among them, DAPG is important since it has a broad spectrum antibacterial, antifungal and antihelminthic activity; (2) secretion of siderophores enabling iron uptake depriving the fungal pathogens in the vicinity; (3) production of low molecular weight metabolites such as hydrocyanic acid (HCN) which inhibits electron transport and hence disruption of energy supply to the cells; (4) production of lytic enzymes such as chitinase, β-1,3 glucanase, protease and lipase which lyse the pathogenic fungal and bacterial cell walls; (5) successfully competes for nutrients against phytopathogens and thereby occupies the colonizing site on root surface and other plant parts and (6) induces systemic resistance in plants by any of the metabolites mentioned above or by the inducting the production of phenyl alanine lyase, antioxidant enzymes such as peroxidase, polyphenol oxidase, superoxide dismutase, catalase, lipoxygenase and ascorbate peroxidase and also by phytoalexins and phenolic compounds in plant cells (Reddy 2013).

#### Biocontrol abilities of rhizobia

Biocontrol is a process through which a living organism limits the growth or propagation of undesired organisms or pathogens. Several rhizobial strains are reported to have the biocontrol properties. Hence, usage of these strains against soil borne pathogens can lead to potential control. The mechanisms of biocontrol by rhizobia include, competition for nutrients (Arora et al. 2001), production of antibiotics (Bardin et al. 2004; Chandra et al. 2007; Deshwal et al. 2003a), production of enzymes to degrade cell walls (Ozkoc and Deliveli 2001) and production of siderophores (Carson et al. 2000; Deshwal et al. 2003b). The production of metabolites such as HCN, phenazines, pyrrolnitrin, viscoinamide and tensin by rhizobia are also reported as other mechanisms (Bhattacharyya and Jha 2012). For example, the strains including *R. leguminosarum* bv. *trifolii, R. leguminosarum* bv. *viciae, R. meliloti*, *R. trifolii, S. meliloti* and *B. japonicum* have been reported to secrete antibiotics and cell-wall degrading enzymes that can inhibit the phytopathogens (Bardin et al. 2004; Chandra et al. 2007; Ozkoc and Deliveli 2001; Shaukat and Siddqui 2003; Siddiqui and Mahmood 2001; Siddiqui et al. 1998, 2000). Rhizobial strains also compete for nutrients by displacing the pathogens. Rhizobia starve the pathogens of available iron by producing high affinity siderophores and thereby limit the growth of the pathogen (Arora et al. 2001). A study on colonization behavior of *P. fluorescens* and *S. meliloti* in alfalfa rhizosphere had sufficiently demonstrated the usage of biocontrol agents to suppress pathogens (Villacieros et al. 2003).

Pathogens that infect okra and sunflower, such as *Macrophomina phaseolina, Rhizoctonia solani* and *Fusarium solani* were shown to be controlled with the usage of *B. japonicum, R. meliloti* and *R. leguminosarum* (Ehteshamul-Haque and Ghaffar 1993; Ozkoc and Deliveli 2001; Shaukat and Siddqui 2003). Some more examples are cyst nematode of potato controlled by *R. etli* strain G12 (Reitz et al. 2000), *Pythium* root rot of sugar beet by *R. leguminosarum* *viciae* (Bardin et al. 2004) white rot disease in *Brassica campestris* by *M. loti* and sheath blight of rice by *R. leguminosarum* bv. *phaseoli* strain RRE6 and bv. *Trifolii* strain ANU843 (Mishra et al. 2006; Chandra et al. 2007). *Xanthomonas maltophilia* in combination with *Mesorhizobium* had been shown to enhance plant growth and productivity in chickpea. This was also been shown to enhance nodule number, nodule biomass and nodule occupancy (Pathak et al. 2007). The incidence of collar rot in chickpea was also shown to reduce by *Pseudomonas* sp. CDB 35 and BWB 21 when co-inoculated with *Rhizobium* sp. IC59 and IC76 (Sindhu and Dadarwal 2001). *Bradyrhizobium* sp. had been shown to control the infection of *M. phaseolina* in peanut, while enhancing seed germination, nodule number and grain yield (Deshwal et al. (2003b). The use of *R. leguminosarum* RPN5, *B. subtilis* sBPR7 and *Pseudomonas* sp. PPR8, isolated from root nodules and rhizosphere of common bean, were shown to be successful against *M. phaseolina*, *F. oxysporum*, *F. solani*, *Sclerotinia sclerotiorum*, *R. solani* and *Colletotrichum* sp. as dual culture or as cell free culture filtrate (Kumar 2012).

#### Induction of plant resistance

The use of PGP strains are reported to trigger the resistance of plants against pathogens. This phenomenon is referred as induced systemic resistance (ISR). In this process, a signal is generated involving jasmonate or ethylene pathway and thus inducing the host plant’s defense response. Various rhizobial species are reported to induce systemic resistance in plants by producing bio-stimulatory agents including *R. etli, R. leguminosarum* bv. *phaseoli* and *R. leguminosarum* bv. *trifolii* (Mishra et al. 2006; Peng et al. 2002; Singh et al. 2006; Yanni et al. 2001). Even individual cellular components of the rhizobium had been shown to induce ISR viz. lipopolysaccharides, flagella, cyclic lipopeptides, homoserine lactones, acetoin and butanediol (Lugtenberg and Kamilova 2009).

## Abiotic stress resistance of rhizobia

The best option for developing stress tolerant crops with minimized production costs and environmental hazards can be the use of PGP microbes as stress relievers. Among them, indigenous and native microbes were more effective and competitive as they are well adapted to the local environments (Mrabet et al. 2005). Rhizobia when used as microbial inoculants have shown many direct and indirect PGP properties including traits for stress are represented in Tables 1 and 2. Rhizobia having some key tolerance mechanism/pathways against certain stress factor such as abiotic stresses, heavy metals and pesticides are required as these are the major constraints for sustainable agriculture. These mechanisms help rhizobia to execute their beneficial PGP traits under stress conditions. The following are some of the resistance mechanisms adopted by rhizobia for their survival and PGP traits for plants under stress conditions.

**Table 1:** Rhizobia and their ability in plant growth promotion (Modified from Ahmed and Kibert, 2014)

Rhizobia	Growth promoting substances produced	References
*Rhizobium*	Siderophores, P-solubilization, IAA, HCN	Abd-Alla (1994a, b), Antoun et al. (1998), Deshwal et al. (2003a), Duhan et al. (1998), Khan et al. (2002), Tank and Saraf (2010)
*Rhizobium* sp.	Growth hormones, IAA, siderophores, HCN, ammonia, exo-polysaccharides	Ahemad and Khan (2009a, 2010a, b, c, 2011a, b, c, 2012a), Joseph et al. (2007), Wani et al.(2007b), Zafar-ul-Hye et al. (2013)
*R. phaseoli* *R. ciceri* *R. meliloti* *R. leguminosarum*	IAA Siderophores Siderophores Cytokinin	Arora et al. (2001), Berraho et al. (1997), Noel et al. (1996), Prabha et al. (2013), Zahir et al. (2010)
*Bradyrhizobium*	Siderophores, IAA, HCN, P-solubilization	Abd-Alla (1994a), Antoun et al. (1998), Deshwal et al. (2003b), Duhan et al. (1998)
*Bradyrhizobium* sp.	IAA, HCN, ammonia, siderophores, exo-polysaccharides	Ahemad and Khan (2011c, d, e, 2012b), Khan et al. (2002), Wani et al. (2007a)
*B. japonicum*	IAA, Siderophores	Shaharoona et al. (2006), Wittenberg et al. (1996)
*Mesorhizobium* sp.	IAA, Siderophores, HCN, Ammonia, exo-polysaccharides, antifungal activity,	Ahemad and Khan (2009b, 2010d, e, 2012c), Ahmad et al. (2008), Khan et al. (2002), Wani et al. (2008a)
*M. ciceri*	IAA, Siderophores	Wani et al. (2007c)

**Table 2:** Attributes of rhizobia exerted against abiotic stress on host plants/in vitro

Rhizobia	Crop species	Screening medium	Growth condition	Remarks		References
Drought stress						
*R. tropici* co-inoculated with *Paenibacillus polymyxa*	Kidney bean	−7, −70 and <−85 kPa	Greenhouse	Enhanced plant height, shoot dry weight and nodule number		Figueiredo et al. (2008)
*Bradyrhizobium* sp.	–	PEG 6000 induced	In vitro and pot culture	Enhanced drought tolerance, IAA and EPS production; nodulation, nodule ARA, nodule N		Uma et al. (2013)
*M. mediterraneum* LILM10	Chickpea	–	Field study	Increased nodule number, shoot dry weight and grain yield Water deficient tolerant strains were also NaCl tolerant.		Romdhane et al. (2009)
*R. elti* (engineered for enhanced trehalose-6-phosphate synthase)	Kidney bean	–	Pot studies	Enhanced nodules, nitrogenase activity and biomass production Higher tolerance than wild type strains		Suárez et al. (2008)
Temperature stress						
*Mesorhizobium* spp.	–	20, 28 and 37 °C	In vitro	Overproduction of 60 kDa protein by all the isolates All the isolates revealed more tolerance to 20 °C than 37 °C.		Rodrigues et al. (2006)
		Heat shock at 60 °C for 15 min; 46 °C for 3 h	In vitro	Variations in the expression of protein profile		
*Rhizobium* sp. DDSS69	–	5 °C	In vitro	Induction of 135 and 119 kDa proteins Variation in the protein profile of stressed and non-stressed cells		Sardesai and Babu (2001)
Salt/osmotic stress						
*M. ciceri* ch-191	Chickpea -salt resistant and sensitive cultivars	50, 75, 100 mM	In vitro	Decreased plant dry weight, nitrogenase activity in sensitive cultivars Less N_2_ fixation inhibition, higher root to shoot ratio, normalized nodule weight and shoot K/Na ratio and reduced foliar accumulation of Na + in resistant cultivars		Tejera et al. (2006)
*M. ciceri* ch-191	–	100–400 mmol NaCl/L	In vitro	Higher tolerance was noticed on 200 mmol/L Altered protein and LPS levels Higher proline accumulation than glutamate		Soussi et al. (2001)
*Acacia* rhizobia. (40 strains)	–	0.4-1.4 M NaCl	In vitro	Presence of small and large plasmids Intracellular accumulation of free glutamate Three rhizobia strains has tolerated 1.4 M NaCl		Gal and Choi (2003)
*M. ciceri, M. mediterraneum and S. medicae*	Chickpea	25 mM NaCl	Glasshouse	M. ciceri enhanced the nodulation and CAT activity Least decrease in nodule protein and SOD activity		Mhadhbi et al. (2004)
Rhizobia strains	Lentil	5.5 Ds m^−1^	Field study	Increased plant biomass, nodule number and nodule dry weight		Islam et al. (2013)
*M. ciceri*, *M. mediterraneum*	Chickpea	Mannitol—50 mM induced	Aerated hydroponic cultures	Maintenance of growth and nitrogen fixing activity Increased antioxidant enzyme activity in nodules		Mhadhbi et al. (2008)
pH stress						
*Mesorhizobium* spp.	–	5, 7 and 9	In vitro	Large range of isolate variation in growth at pH 7/9 and others at pH 5/7		Rodrigues et al. (2006)

Abiotic stresses, such as drought, extremes of temperature, soil salinity, acidity, alkalinity and heavy metals causes severe yield loss. The response of legumes to various stresses depends on the host plant reaction, but this reaction can be influenced by the rhizobia and the process of symbiosis (Yang et al. 2009). The role of microorganisms in adaptation of crops to various abiotic stresses is reviewed by Grover et al. (2010). There are comprehensive reviews on tolerance and nodulating capacity of *Rhizobium* and *Bradyrhizobium* to soil acidity, salinity, alkalinity, temperature and osmotic stress conditions (Graham 1992; Kulkarni and Nautiyal 2000). A list of related studies that are in Table 2.

### Extremes of temperature

Global warming and the resultant climate change are expected to cause land degradation with salinization, increase the drought episodes and desertification (USDA 2012). High temperatures lead to increased drought intensity, due to enhanced transpirational water loss. This can lead to reduction in nodule number, rhizobial growth, rate of colonization and infectious events, and can lead to delay in nodulation or restrict the nodule to the subsurface region. This phenomena was observed in alfalfa plants grown in a desert environment of California (USA) forming fewer nodules in the top 5 cm soil horizon, while extensively nodulating below this depth (Munns et al. 1979).

The optimum temperature for rhizobial growth is 28–31 °C, while many of them are unable to grow beyond 37 °C. Rhizobia isolated from hot and dry environments of the Sahel Savannah are reported to tolerate temperature up to 45 °C, but they were found to lose their infectiveness (Eaglesham and Ayanaba 1984; Hartel and Alexander 1984; Karanja and Wood 1988). Similarly, a heat treatment of 35 and 37 °C to *R. phaseoli* was found to cause loss of melanin synthesis plasmid DNA and symbiotic properties (Beltra et al. 1988). In contrast, at 35 and 38 °C, *R. leguminosarum* bv. *phaseoli* was found to be infective and formed nodules in *P. vulgaris*, but these nodules were found to remain ineffective (Hungria and Franco 1993). Nevertheless, heat-tolerant, actively nodulating and N_2_ fixing *Rhizobium* strains have been identified. Adaptation of microorganisms to stress is a complex regulatory process, as it involves the use of proteins and lipopolysaccharide (LPS) with the up-regulation of an array of genes. Upon exposing the wild and heat resistant *Rhizobium* sp. to 30 and 43 °C, changes in the cell surface including extracellular polymeric substances/exo polysaccharides (EPS), LPS and proteins had been demonstrated (Nandal et al. 2005). Michiels et al. (1994) reported first about the presence of a large set of small heat shock proteins in *Rhizobium* sp. They observed the expression of eight heat shock proteins in heat-sensitive strains, where as it was only two in the heat-resistant strains, which indicates that the heat shock proteins also play key roles in normal cell growth. Münchbach et al. (1999) reported 12 small heat shock proteins in *B. japonicum* and classified them into Class A—sHsp similar to *Escherichia coli* *IbpA* and *IbpB*, and Class B—sHsps similar to sHsps from other prokaryotes and eukaryotes. Among the sHsp family, 13 genes for small heat shock proteins was detected on *B. japonicum* (Han et al. 2008). Among these regulatory systems, chaperones such as *DnaK*–*DnaJ* and *GroEL*–*GroES* are the key components of heat shock or stress response. These chaperones help on hydrophobic domains of the target protein to regain their native structure since they get denatured upon stress (Hartl and Hayer-Hartl 2009). Alexandre and Oliveira (2011) reported 53 strains of *Mesorhizobium* sp. for heat stress and shock protein production by chaperone analysis, which revealed the increased transcripts of *dnaK* and *groESL*. They also observed a higher induction of chaperone genes in heat-tolerant isolates than in heat-sensitive isolates of the same species, however, such phenomenon was not found to express during cold stress conditions. This indicates that although the chaperones are heat shock proteins, their gene expression is stress specific.

Plants have various mechanisms for drought tolerance including drought-escapism, dehydration postponement and dehydration tolerance (Turner et al. 2000). Plants generally overexpress zeatin for delayed leaf senescence as a drought tolerance mechanism. Alfalfa plants inoculated with engineered strains of *S. meliloti* with *ipt* gene showed elevated zeatin concentrations and antioxidant enzymes in their leaves and survived better under severe drought conditions (Xu et al. 2012). Vanderlinde et al. (2010) noticed the production of EPS as another tolerance mechanism. Intra species difference in competitive efficiency was demonstrated by Krasova-Wade et al. (2006) in which *Bradyrhizobium* ORS 3257 was found to compete their best under favorable water conditions while *Bradyrhizobium* ORS 3260 was the best under limited water conditions.

### Salinity

Soil salinity is one of the production constraints in the arid and semi-arid tropics world-wide, and about 40 % of the world’s land surface is affected by salinity-related problems (Zhan et al. 1991). Salinity decreases the nutrition uptake of plants, particularly P, due to their binding with Ca ions in salt-stressed soils. It is also known that higher concentration of ions (Na^+^, Cl^−^, SO_4_^2−^) in saline soils gets accumulated in the plant cells and inactivate enzymes that inhibits protein synthesis and photosynthesis (Serraj et al. 1994; Zhu 2001). Salinity affects bacterial infection process (by decreasing the number and the deformation of root hairs), nodule growth and functioning (by limiting the nutrient supply via photosynthesis products and oxygen consumption) and BNF (by reducing the nodule metabolism, leghemoglobin content and atmospheric nitrogen diffusion).

Rhizobial species are known to vary in their salt sensitivity. Some of them are categorized as salt tolerant, such as *R. meliloti* (Zhang et al. 1991), *R. fredii* (Yelton et al. 1983), *Rhizobium* sp. from *Acacia senegal*, *Prosopis chilensis* (Zahran et al. 1994) and *Vigna unguiculata* (Mpepereki and Makoneses 1997), chickpea, soybean (El Sheikh and Wood 1990), and pigeonpea (Subbarao et al. 1990) whereas others as salt sensitive such as *R. leguminosarum* (Chein et al. 1992). The existence of a high degree of phenotypic and genotypic diversity in *Sinorhizobium* populations sampled from marginal soils of arid and semi-arid regions of Morocco has been demonstrated recently (Thami-Alami et al. 2010). It was also observed that these salt tolerant isolates also turned out to be water stress tolerant. The effect of salt stress on halotolerant rhizobia by their LPS (Lloret et al. 1995; Zahran et al. 1994), protein profiles (Saxena et al. 1996) and exopolysaccharide (Lloret et al. 1998) have been studied. Large variability in the efficiency of host plant and rhizobial strains on BNF under salinity had been reported (Jebara et al. 2001; Aouani et al. 1998).

Salinity generates negative osmotic potential that lowers the soil water potential. Similar to plants, rhizobia also produce many group of metabolites called compatible solutes [trehalose, N-acetylglutaminylglutamine amide (NAGGN) and glutamate], osmoprotectants [betaine, glycine-betaine, proline-betaine, glucans, trehalose, sucrose, ectoine, 3-dimethylsulfoniopropionate (3-dimethylpropiothetin or DMSP), 2-dimethylsulfonioacetate (2-dimethylthetin or DMSA)] and pipecolic acid and cations [calcium, potassium] as tolerance mechanism (Chen 2011; Streeter 2003; Sugawara et al. 2010). Salt tolerance mechanisms involve several gene families which have been reported largely in *S. meliloti* followed by *R. etli, R. tropici, Rhizobium* sp., *S. fredii* and *B. japonicum*. The identified gene families includes betaine (*betS*/*betABCI*/*hutWXV*) (Boscari et al. 2002), glycine-betaine (*AraC*) and proline-betaine (*betS*/*prb*) (Boscari et al. 2004; Payakapong et al. 2006; Alloing et al. 2006), glucans (*ndvABCD*), sucrose (*zwf*) and trehalose (*zwf*) (Chen et al. 2002; Jenson et al. 2002; Barra et al. 2003), cation efflux (*phaA2*/*phaD2*/*phaF2*/*phaG2*) (Jiang et al. 2004) and *rpoH2* (Tittabutr et al. 2006), *ntrY*, *ntrX*, *greA*, *alaS*, *dnaJ*, *nifS*, *noeJ*, *kup* (Nogales et al. 2002), *omp*10, *relA*, *greA* and *nuoL* (Wei et al. 2004). Osmolyte production depends not only on type of stress, but also on degree of stress. It was reported in *S. meliloti* that at lower level of salt concentration glutamate accumulates; at higher levels, glutamate and NAGGN accumulates, whereas at extremely higher concentrations, all the three osmolytes, glutamate, NAGGN, and trehalose accumulates (Smith et al. 1994). Osmoprotectants, the compatible solutes/osmolytes, also play a dual role as evidenced in *S. meliloti* by proline-betaine which serves as both osmoprotectant (under high osmotic stress) and energy source (under low osmotic stress) (Miller-Williams et al. 2006).

### Soil acidity

Soil acidity (low pH) is yet another abiotic stress that affects plant growth and cause crop failures which might be due to high concentration of protons and low concentration of calcium and phosphate in acidic soils. Low survival and poor growth of rhizobia and inhibition of initiation and formation of root nodules are the important responses that lead to the failure of rhizobia–legume symbiosis in acid soils (Richardson et al. 1988). The addition of lime on acid soils has been followed as a common practice to raise the soil pH creating a favorable conditions for the growth and survival of root nodule bacteria (Watkin et al. 1997). Graham et al. (1994) proposed some strains of *Rhizobium, Azorhizobium* and *Bradyrhizobium* to be low pH tolerant. Tolerance to acidity by rhizobia was correlated with the production of extracellular polysaccharide or polyamines/glutamate concentration in the cell. Muglia et al. (2007) highlighted the role of glutathione, a tripeptide for the growth of *R. tropici* under low pH conditions. Watkin et al. (2003) reported the ability of acid tolerant *R. leguminosarum* bv. *trifolii* in accumulating higher level of potassium and phosphorous than an acid sensitive strain. The effect of acid shock on *S. meliloti* 1021 was analyzed via oligo-based whole genome microarrays which demonstrated that within 20 min of the shock, the cells had started to respond by either up-regulating or down-regulating the specific genes of various cellular functions or hypothetical proteins of unknown functions (Hellweg et al. 2009). In a re-vegetation program on acidic soils, *Bradyrhizobium* sp. was found to enhance the nodule number and plant growth when six shrubby legumes, such as *Cytisus balansae, C. multiflorus, C. scoparius, C. striatus, Genista hystrix* and *Retama sphaerocarpa,* were inoculated with (Rodríguez-Echevarría and Pérez-Fernández 2005).

### Heavy metal resistance of rhizobia

Pollution of the biosphere by the toxic metals had increased dramatically since the beginning of industrial revolution by the dumping of solid wastes and the use of industrial waste waters for irrigation. Ever increasing demand for lands, forced the farmers to use contaminated sites for crop cultivation. Heavy metals are the key pollutants causing serious illness to plants, ecosystem and humans by their non-degradable nature. For the reclamation and removal of heavy metals, phytoremediation is suggested to be practiced as it preserves natural soil properties and microbial biomass (Gianfreda and Rao 2004). The use of microorganisms such as *Bacillus* sp., *Pseudomonas* sp., *Azotobacter* sp., *Enterobacter* sp., and *Rhizobium* sp. were also proposed to speed up the phytoremediation process had been reviewed in detail by Ma et al. (2011).

Rhizobia multiply slowly in soil until they infect a compatible host. Rapid growth of rhizobia occurs only after successful infection by a single cell and formation of a nitrogen-fixing nodule on the host-root which consists of over 108 cycles of bacterial progeny (Downie, 1997). In heavy metal contaminated sites, after the successful establishment of symbiosis with the host plant, the heavy metals tend to accumulate in the nodules. This would be an alternative and less expensive mode to (1) remove heavy metal from soil (2) enhance soil and plant health with an enhanced nitrogen fixation and other plant growth promoting pathways and (3) help to grow heavy metal-free plant components in any contaminated site contributing to food and nutritional security. However, despite demonstrating the extent of benefits through the use of PGPR in remediation of contaminated sites, there had been very few field studies while most of the successful studies are either from greenhouse or growth chambers (Lucy et al. 2004).

Effects of heavy metals on growth, abundance, morphology and physiology of various strains of *R. leguminosarum* have been well documented (Castro et al. 1997; Chaudhary et al. 2004; Chaudri et al. 2000; Lakzian et al. 2002; Smith 1997). Continuous exposure to heavy metals leads the viable bacterial cells not only to transform into a non-viable form, but also adversely affects the genetic diversity and nodulation of the host plants (Paton et al. 1997; Hirsch et al. (1993). Reductions in bacterial counts of *Rhizobium* sp. have been reported with the increasing concentrations of heavy metals such as Cu, Zn and Pb, either sole or in combinations, and variations in the expression of symbiotic genes including *nod* genes (Stan et al. 2011).

Studies on effectiveness of rhizobia isolated from long-term contaminated sites (over 40 years) and un-polluted sites revealed that only 15 % of the active isolates were effective in polluted sites while 94 % of them were from un-polluted sites. A great diversity in terms of plasmid types has been observed in isolates of un-polluted soil than the isolates from polluted soils. In addition, the dominant plasmid groups present in un-polluted soils were found to be absent in isolates of polluted soils and vice versa (Castro et al. 1997). However, these negative impacts had been specific to exposure time and metal type. This hypothesis had been demonstrated by Carrasco et al. (2005), who isolated 41 heavy metal resistant isolates of rhizoibia from a total of 100 isolates in a site contaminated since 6 years by a toxic spill. Genetic diversity among the rhizobial population had also been observed through differences in size of *nod*C fragment, heavy metal resistance and symbiotic properties. Some strains had been observed to have a broad spectrum resistance and therefore are symbiotically effective even under combined stress conditions. Changes in physiology were found to lead to the variations in protein profiles that serve as a marker for stress response analysis in *R. leguminosarum* bv. *viciae* isolated from heavy metal polluted sites (Pereira et al. 2006a).

Similar to the non-nodulating bacterial species, rhizobia also has its own features such as EPS and LPS for influencing heavy metal resistance. EPS are biopolymers that possess negatively charged ligands which instantly form complexes with metal ions through electrostatic interactions (Liu et al. 2001; Sutherland 2001). EPS from *R. etli* (strain M4), isolated from an acid mine drainage, was shown to impact ecosystem near a manganese mine in Northern Australia (Foster et al. 2000; Pulsawat et al. 2003). Lakzian et al. (2002) identified that plasmids are the major contributing factor for this as highly tolerant strains were noticed to have 6–9 plasmids whereas moderately tolerant strains have only three plasmids. However, an alternate view was reported by Pereira et al. (2006b) on cadmium (Cd) resistance as he found similar number (a maximum of four) plasmids in all the tolerant, moderately tolerant and sensitive isolates. Pereira et al. (2006b) also observed that some highly tolerant strains have had no plasmids and therefore had concluded that, the heavy metal resistance may be related to the plasmids, but there could also be some other mechanisms conferring metal resistance. This study also reported that the concentration of intracellular Cd varies within the groups, where highly tolerant strains have higher quantity. Reports of Figueira et al. (2005), Purchase et al. (1997), and Purchase and Miles (2001), are also support this view.

Natural resistance is not sufficient when soils are contaminated heavily and for a longer period. Use of recombinant rhizobia could play a major role in remediation measures. Microorganisms equipped with high metal-binding capacity through metallothionins for enhancing the tolerance, sequestration of heavy metals have been widely exploited. Metallothionins (MTs) are the low-molecular weight, cysteine-rich, metal binding proteins produced by higher organisms (Kagi 1991). Sriprang et al. (2002) engineered the expression of tetrameric human MTL-4 gene under *nifH* and *nolB* promoters in *M. huakuii* subsp. *rengei* B3 that had been shown to establish symbiosis with *Astragalus sinicus* in Cd-polluted soils and to enhance Cd uptake by twofolds.

Phytochelatins (PCs), a naturally occurring peptide, having metal binding properties and found in a variety of plants and microorganisms (Cobbett 2000), can accumulate higher concentration of heavy metals than MTs due to their unique structural features (Mehra and Mulchandani 1995). Sriprang et al. (2003) transformed PCs gene from *Arabidopsis thaliana* (*AtPCS*) to *M. huakuii* subsp. *rengei* B3 strain for enhancing the adsorption of heavy metals. They found that the free-living state of the recombinant bacterium had a higher Cd accumulation while the symbiotic state had a higher accumulation of Cu and As than Cd and Zn (Ike et al. 2008).

It is necessary to isolate and study the native rhizobial strains from heavy metals contaminated soils, to identify the potential of rhizobium–legume symbiosis of particular strain for the remediation of the affected area. Such studies with their contribution are presented in Table 3. Rhizobia, such as *R. fredii*, *R. meliloti*, *R. etli*, *R. leguminosarum* bv. *viceae*, *R. leguminosarum* bv. *trifolii*, *Bradyrhizobium* sp. and *B.* *japonicum* had been evaluated for heavy metal resistance and of which *R. fredii* and *R. meliloti* alone were found to exhibit higher metal tolerance against Tellurium (Te) and Selenium (Se) (Kinkle et al. 1994). Nonnoi et al. (2012) demonstrated differences in the heavy metal resistance spectrum of *S. medicae* and *R. leguminosarum* bv. *trifolii* strains isolated from mercury-contaminated soils. Heavy metals are reported to cause harm not only to benefiting microbes, but also to host plants. Paudyal et al. (2007) reported the negative effect of heavy metals such as Al, Fe and Mo on two *Rhizobium* strains and their symbiotic efficiency on host plants. Chaudri et al. (2000) observed greatly reduced symbiosis of *R. leguminosarum* bv. *viciae* with pea and *R. leguminosarum* bv. *trifolii* with white clover under Zn toxicity as a consequence of reduced numbers of free-living rhizobia in the soil indirectly affecting N fixation and Zn phytotoxicity. Severe yellowing of plants, small leaves, lack of nodules and reduced rhizobial counts has also been observed as the symptoms of heavy metal toxicity in these toxicity affected plants.

**Table 3:** Rhizobia and their effects on host plant/in vitro at metal stress conditions

Rhizobia	Crop species	Heavy metal stress	Growth condition	Remarks	References
*R. leguminosarum* bv. *trifolii* NZP561	–	Cd	In vitro	Sequestration of Cd	Robinson et al. (2001)
*Bradyrhizobium* RM8	Green gram	Ni, Zn	Pot experiments	Enhanced growth performance	Wani et al. (2007c)
*Rhizobium* sp.	Hyacinth bean	Co, Cu, Zn, Cd	Pot experiments and field conditions	Greater HM accumulation in nodules than in roots and shoots	Younis (2007)
*Mesorhizobium* RC3	Chickpea	Cr	Pot experiments	Increased growth, nodulation, chlorophyll, leghaemoglobin, nitrogen content, seed protein and seed yield	Wani et al. (2008b)
*M. metallidurans* sp. nov.	–	Cd, Zn	In vitro	Heavy metal resistance	Vidal et al. (2009)
*R. leguminosarum*	Maize	Pb	Pot experiments	Enhanced plant growth and biomass	Hadi and Bano (2010)
*S. meliloti*	Black medic	Cu	Pot experiments	Enhanced biomass production	Fan et al. (2011)
*Rhizobium* RL9	Lentil	Pb, Ni	Pot experiments	Increased growth, nodulation, chlorophyll, leghaemoglobin, nitrogen, seed protein and seed yield	Wani and Khan (2012, 2013)

Besides nitrogen fixation and heavy metal resistance, some rhizobia exhibit PGP traits under contaminated conditions as reported in soybean cv. Curringa and its rhizobial symbiont *B. japonicum* at higher arsenic (As) concentrations (Reichman 2007). Guo and Chi (2014) reported cadmium (Cd) tolerant *Bradyrhizobium* sp . to exhibit several PGP traits including synthesis of IAA, ACC deaminase, siderophores, increased shoot dry weights and high level accumulation of Cd in roots of *Lolium multiflorum* than in un-inoculated control. They also reported that the strain enhanced the extractable Cd concentrations in the rhizosphere, whereas it decreased the Cd accumulation in root and shoot of *G. max* by increasing Fe availability.

Huang et al. (2005) reported developing a multi-process phytoremediation system (MPPS) for petroleum hydrocarbons. This employs the use of both PGP bacteria and specific contaminant-degrading bacteria which metabolize the contaminants into non-toxic substances/readily available compounds while the role of PGP bacteria is still prompting plant growth and increasing the plant tolerance to pollutants. MPPS has also the potential for deployment to enhance rhizobium-host symbiosis and plant growth at heavy metal contaminated sites.

### Pesticide tolerance of rhizobia

Pesticide accumulation in soils beyond the recommended level occurs either by consistently repeated application or their slow degradation rate. It affects plant growth by altering plant root’s architecture, number of root sites for rhizobial infection, transformation of ammonia into nitrates, transformation of microbial compounds to plants and vice versa. Besides this, growth and activity of free-living or endophytic nitrogen fixing bacteria has also been affected (Mathur 1999). Several studies have documented the effects of various pesticides on the reduction of microbial diversity and density on various soil types (El Abyad and Abou-Taleb 1985; Moorma 1988; Martinez-Toledo et al. 1996). Numerous microorganisms have the capacity to degrade the pesticides by the action of degradative genes in plasmids/transposons/chromosomes (Kumar et al. 1996). The influence of broad-spectrum of pesticides on the functional attributes of rhizobia and their tolerance to pesticides are reported in Table 4. From the literature survey, it was recognized that none of the rhizobia are found to have pesticide tolerance under field conditions. So research on isolating, identifying and characterizing such resistant rhizobia needs to be vigorously pursued as such rhizobia are very much needed considering the quantum of pesticide residue generated currently.

**Table 4:** Rhizobia and their beneficial attributes exerted on host plant/in vitro at pesticide stress conditions

Rhizobia	Crop species	Pesticides	Concentrations	Condition	Remarks	References
		*Herbicides*				
*Rhizobium* MRP1 *Rhizobium* MRL3 *Mesorhizobium* MRC4	Pea Lentil Chickpea	Quizalafop-p-ethyl	40, 80 and 120 µg/kg soil	Pot experiments	Enhanced biomass, nodulation, leghaemoglobin content, root and shoot N, root and shoot P, seed yield and seed protein	Ahemad and Khan (2009a, 2010b, d)
		Clodinafop	400, 800 and 1,200 µg/kg soil			
*Rhizobium* MRP1 *Rhizobium* MRL3 *Mesorhizobium* MRC4 *Bradyrhizobium* MRM6	–	Metribuzin	850, 1,700 and 2,550 µg/L	In vitro	Concentration-dependent progressive decline in PGP substances except exo-polysaccharides	Ahemad and Khan (2011a, b, 2012a, c)
		Glyphosate	1,444, 2,888 and 4,332 µg/L			
*R. leguminosarum* RCR 1045	–	Terbuthylazine	4, 8, 16, 32 and 64 mg/L	In vitro	Growth decline was in the order of Terbuthylazine > Prometryn > Simazine	Singh and Wright (2002)
		Simazine	4.3, 8.6, 17.2, 34.4 and 68.8 mg/L			
		Prometryn	4, 8, 16, 32 and 64 mg/L			
		Bentazon	4.1, 8.2, 16.4, 32.8, 65.6 mg/L		No adverse effects on growth	
		*Insecticides*				
*Rhizobium* MRL3 *R. leguminosarum* MRP1 *Mesorhizobium* MRC4	Chickpea, Pea, Lentil	Fipronil	200, 400, and 600 mg/kg soil	Pot experiments	Enhanced the biomass, nodulation, leghaemoglobin content, root and shoot N, root and shoot P, seed yield and seed protein	Ahemad and Khan (2009b, 2010a, 2011f)
		Pyriproxyfen	1,300, 2,600, and 3,900 mg/kg soil			
*Rhizobium* MRP1 *Rhizobium* MRL3 *Mesorhizobium* MRC4 *Bradyrhizobium* MRM6	–	Imidacloprid	100, 200 and 300 µg/L	In vitro	Concentration-dependent progressive decline in PGP substances except exo-polysaccharides	Ahemad and Khan (2011a, b, 2012a, c)
		Thiamethoxam	25, 50 and 75 µg/L			
		*Fungicides*				
*Rhizobium* MRP1 *Rhizobium* MRL3 *Mesorhizobium* MRC4 *Bradyrhizobium* MRM6	–	Hexaconazole	40, 80 and 120 µg/L	In vitro	Concentration-dependent progressive decline in PGP substances except exo-polysaccharides	Ahemad and Khan (2011a, b, 2012a,c)
		Metalaxyl	1,500, 3,000 and 4,500 µg/L			
		Kitazin	96, 192 and 288 µg/L			
*Rhizobium* MRP1	Pea	Tebuconazole	100, 200 and 300 µg/L	In vitro	Concentration-dependent progressive decline in PGP substances except exo-polysaccharides, HCN and ammonia	Ahemad and Khan (2011d)
			100, 200 and 300 µg/kg soil	Pot experiments	Enhanced the biomass, nodulation, leghaemoglobin content, root and shoot N, root and shoot P, seed yield and seed protein	

## Synergistic effects of rhizobial co-inoculation

The in-consistency of beneficial results of microbial use, when single microbe was used in the field application, have brought an emphasis on co-inoculation of microbes (Bashan and de Bashan 2005). Certain specific co-inoculation causes synergy by functioning as helper bacteria to improve the performance of the other bacteria. Therefore in such co-inoculations, the combination of PGP bacteria, rhizobia and the host genotype has to be selected after extensively careful evaluations (Remans et al. 2007, 2008). A range of PGP microbes can be used with rhizobium that not only improves legume growth and yield but also cost effective and efficient.

*Azospirillum*, a free living diazotroph*, Azotobacter*, *Bacillus*, *Psuedomonas*, *Serretia,* and *Enterobacter* are some of the genera that are successfully used with rhizobium as co-inoculants. *Azospirillum*, was found to enhance growth and yield of several leguminous crops upon inoculation (Roseline et al. 2008). Improved nodulation was found when *A. lipoferum* and *R. leguminosarum* bv. *trifolii* were co-inoculated in white clovers (Tchebotar et al. 1998), pigeonpea and chickpea (Deanand et al. 2002). It was found that *Azospirillum* can increase the infection site providing a space for *Rhizobium* resulting in higher nodule formation (Tchebolas et al. 1988). Co-inoculation with *Azospirillum* and *Rhizobium* were shown to increase phytohormones, vitamins and siderophore production (Cassan et al. 2009; Dardanelli et al. 2008). Co-inoculation of common bean with *Azospirillum*-*Rhizobium* was also shown to increase the fixed nitrogen quantity (Reman et al. 2008). *Azotobacter* was found to be a potential co-inoculant with rhizobium that enhanced the production of phytohormones and vitamins and increase the nodulation (Akhtar et al. 2012; Chandra and Pareek 2002; Dashadi et al. 2011; Qureshi et al. 2009). *Bacillus* sp. was also been reported to induce the PGP ability, yield (Mishra et al. 2009; Ahmad et al. 2008) and uptake of nutrients (Stajkovic et al. 2011) including phosphorous (Singh et al. 2011). Significant increase in weight of the root and seed yield of chickpea were reported upon inoculation of *Rhizobium* with *B. subtilis* OSU-142 and *B. megaterium* M-3 (Elkoca et al. 2008). Enhanced nodulation and nitrogen fixation was noticed upon inoculation of *Bacillus* and *Azospirillum* sp. along with rhizobial inoculants in pigeonpea (Rajendran et al. 2008; Roseline et al. 2008). Interaction between *Streptomyces* *lydius WYEC108* and *Rhizobium* of pea were shown to promote growth of the plant (Tokala et al. 2002) including nodule number and growth, probably by the root and nodule colonization of *Streptomyces*. *Enterobacter* is another most abundant PGP bacteria that increased the yield of nodules on green gram when co-inoculated with *Bradyrhizobium sp*. (Gupta et al. 1998). When *R. tropici* CIAT899 was co-inoculated with *C*. *balustinum* Aur9 it resulted in increased root hair formation and infection sites leading to early nodule development and increased nodule formation (Estevez et al. 2009). Similar result was obtained when *Medicago truncatula* cv. *Caliph* was co-inoculated with *Pseudomonas fluorescens* WSM3457 and *Ensifer* (*Sinorhizobium*) *medicae* WSM419 (Fox et al. 2011). Recently, it was found that nodulation, root and shoot dry weight, grain and straw yield, nitrogen and phosphorus uptake were significantly increased in chickpea upon co-inoculation with *Mesorhizobium* sp. and *P. aeruginosa* (Verma et al. 2013). Similar plant growth effects along with the antagonistic activities against *F. oxysporum* and *R. solani* has been observed on chickpea by co-inoculation of *Mesorhizobium, Azotobacter chroococcum, P. aeruginosa* and *Trichoderma harzianum* (Verma et al. 2014). Mehboob et al. (2013) had a recent detailed review highlighting the effects of co-inoculation of rhizobia with various rhizospheric bacteria. Although there are many combinations of bacteria were explored for use, still there is a need for an advanced comprehensive research in the area.

## Conclusion

Rhizosphere is a unique niche that provides habitation and nutrition to PGP microorganisms. In turn, these microorganisms produce multiple benefits of induced plant growth, defense against diseases and survival under stress with many other unknown benefits. The present review documents the potential of PGP rhizobia and highlights the unique properties of plant growth induction, defense pathways and the resistance spectrum available against various abiotic stresses on a variety of agricultural crops. However, the extent of success in realizing the benefits of PGP tends to diminish as it moves from laboratory to greenhouse and to fields, which reflects the scarcity of research on the beneficial effects of PGP microbes under field conditions. Therefore, generation of comprehensive knowledge on screening strategies and intense selection of best rhizobacterial strain for rhizosphere competence and survival is the current need to enhance the field level successes. Identification of such potential rhizobial strains and developing a robust technology for the use by smallholder farmers is still in its infancy. Thus, additional comprehensive research to exploit the potential of PGP rhizobia would provide for expansion of this research area, commercialization and improve sustainability in agricultural production.
